# CYCLoPs: A Comprehensive Database Constructed from Automated Analysis of Protein Abundance and Subcellular Localization Patterns in *Saccharomyces cerevisiae*

**DOI:** 10.1534/g3.115.017830

**Published:** 2015-06-09

**Authors:** Judice L. Y. Koh, Yolanda T. Chong, Helena Friesen, Alan Moses, Charles Boone, Brenda J. Andrews, Jason Moffat

**Affiliations:** * The Donnelly Centre, University of Toronto, Toronto, Ontario, Canada, M5S3E1; † Department of Cell & Systems Biology, University of Toronto, Toronto, Ontario, Canada, M5S3E1; ‡ Department of Molecular Genetics, University of Toronto, Toronto, Ontario, Canada, M5S3E1

**Keywords:** GFP, subcellular localization, abundance, images, microscopy

## Abstract

Changes in protein subcellular localization and abundance are central to biological regulation in eukaryotic cells. Quantitative measures of protein dynamics *in vivo* are therefore highly useful for elucidating specific regulatory pathways. Using a combinatorial approach of yeast synthetic genetic array technology, high-content screening, and machine learning classifiers, we developed an automated platform to characterize protein localization and abundance patterns from images of log phase cells from the open-reading frame−green fluorescent protein collection in the budding yeast, *Saccharomyces cerevisiae*. For each protein, we produced quantitative profiles of localization scores for 16 subcellular compartments at single-cell resolution to trace proteome-wide relocalization in conditions over time. We generated a collection of ∼300,000 micrographs, comprising more than 20 million cells and ∼9 billion quantitative measurements. The images depict the localization and abundance dynamics of more than 4000 proteins under two chemical treatments and in a selected mutant background. Here, we describe CYCLoPs (Collection of Yeast Cells Localization Patterns), a web database resource that provides a central platform for housing and analyzing our yeast proteome dynamics datasets at the single cell level. CYCLoPs version 1.0 is available at http://cyclops.ccbr.utoronto.ca. CYCLoPs will provide a valuable resource for the yeast and eukaryotic cell biology communities and will be updated as new experiments become available.

During the past decade, proteome-wide screens in a variety of experimental systems have begun to elucidate how protein networks are organized in eukaryotic cells (*e.g.*, Ghaemmaghami *et al.* 2003; Huh *et al.* 2003; Newman *et al.* 2006; Nagaraj *et al.* 2012; Kulak *et al.* 2014). We have developed a method for integrating systematic genetics, high-throughput microscopy, image analysis and pattern classification into an automated data acquisition and analysis platform for cell biological screens in budding yeast (Chong *et al.* 2015). Our screening pipeline makes use of the yeast GFP (green fluorescent protein) collection, which consists of a series of haploid yeast strains in which each open-reading frame (ORF) is individually tagged, generating a full-length protein with a COOH-terminus GFP fusion, whose expression is driven by the endogenous ORF promoter (Huh *et al.* 2003). We worked with the set of 4144 strains from the original collection previously annotated as having a visible GFP signal and representing ∼71% of the yeast proteome. We used this collection to measure the subcellular localization and abundance of yeast proteins at the single-cell level in several conditions in time courses of up to 11 hr (Chong *et al.* 2015).

A number of existing databases present images of yeast cells from large-scale studies. Some of these studies assess phenotypes associated with analysis of a small number of morphologic characteristics or markers in a collection of mutants. Databases that house this type of data include SCMD (Saito *et al.* 2004) and PhenoM (Jin *et al.* 2012). Other databases present images of a collection of GFP (or otherwise)-tagged proteins in one or a few genetic backgrounds or conditions. Examples of this type include the Yeast GFP Fusion Localization Database, YGFP (Huh *et al.* 2003), the Yeast Protein Localization Database, YPL (Kals *et al.* 2005), Organelle DB (Wiwatwattana *et al.* 2007), the Yeast Resource Center, YRC (Riffle and Davis 2010), the Localization and Quantitation Atlas of the Yeast Proteome, LOQATE (Breker *et al.* 2013), and Cellbase (Dénervaud *et al.* 2013). Several of these databases present visually annotated protein localizations together with the images (YGFP, YPL, LOQATE), two quantify protein abundance (LOQATE, Cellbase), and one assesses the probability of each cell displaying any mixture of six main spatial patterns (Cellbase); however, none of them computationally defines a localization for each GFP protein.

To enable easy access of our image compendium of subcellular localization and abundance profiles to the research community, we developed a web-accessible database called CYCLoPs (Collection of Yeast Cells and Localization Patterns) that allows retrieval and visualization of yeast cell images and permits queries of the subcellular localization and abundance profiles of the yeast proteome for each genetic or chemical perturbation in our survey. CYCLoPs currently contains a total of 330,248 images from three wild-type screens, three screens with a strain deleted for the gene encoding the conserved lysine deacetylase Rpd3, and time courses of two chemical treatments (hydroxyurea and rapamycin; Table 1). CYCLoPs differs from existing databases in a number of ways: (1) whereas other databases provide searchable localization assignments for proteins that had been assessed visually, CYCLoPs contains computationally derived quantitative localization and abundance profiles; (2) CYCLoPs provides a searchable web graphical interface for proteins with localization and/or abundance changes of interest, which reflects the proteome flux in response to varying environmental cues and genetic backgrounds; (3) the subcellular localization data hosted on CYCLoPs were determined directly from the morphologic features of the cells and accommodate the reality that many proteins localize to multiple locations; and (4) CYCLoPs provides localization and abundance profiles for individual cells screened, thus enabling analysis at the single-cell level.

**Table 1:** Summary statistics for 18 cell biological screens whose results are housed in CYCLoPs

Screen	Condition	Time Course	Control	No. of Micrographs	No. of Cells
WT1	*wild-type*	−	−	17,908	1,107,029
WT2	*wild-type*	−	−	18,429	1,187,761
WT3	*wild-type*	−	−	17,908	1,102,945
HU80	*hydroxyurea*	80 min	WT3	18,428	1,158,646
HU120	*hydroxyurea*	120 min	WT3	18,432	1,540,635
HU180	*hydroxyurea*	180 min	WT3	18,432	1,679,998
RAP60	*rapamycin*	60 min	WT3	18,432	1,150,818
RAP140	*rapamycin*	140 min	WT3	18,428	1,607,301
RAP220	*rapamycin*	220 min	WT3	18,432	1,782,059
RAP300	*rapamycin*	300 min	WT3	18,428	2,205,984
RAP380	*rapamycin*	380 min	WT3	18,001	2,360,608
RAP460	*rapamycin*	460 min	WT3	18,426	1,798,178
RAP540	*rapamycin*	540 min	WT3	18,432	2,148,814
RAP620	*rapamycin*	620 min	WT3	18,432	1,844,265
RAP700	*rapamycin*	700 min	WT3	18,428	2,143,449
rpd3Δ_1	*rpd3 knockout*	−	WT3	18,424	1,140,087
rpd3Δ_2	*rpd3 knockout*	−	WT3	18,424	987,083
rpd3Δ_3	*rpd3 knockout*	−	WT3	18,424	933,041
Total				330,248	27,878,701

## Results and Discussion

### Microscopy data acquisition and analysis

Details of the experimental approach are described in Chong *et al.* (2015). In summary, the yeast synthetic genetic array protocol (Tong *et al.* 2001) was coupled with a high-content microscopy platform to image an arrayed collection of 4144 arrayed strains carrying a C-terminal fusion of GFP to each ORF (Huh *et al.* 2003) and expressing a tdTomato fluorescent protein from the constitutive *RPL39* promoter. The tdTomato protein is localized to the cytoplasm and allows identification of cell boundaries during automated imaging. Micrographs were acquired using a high-throughput spinning-disc confocal microscope (Opera; PerkinElmer). Eight images were acquired from each strain, four in the red channel and four in the green channel, and analyzed via the CellProfiler, version 5811 (Carpenter *et al.* 2006). On average, 84 cells were captured from each micrograph; between 900,000 and 2.4 million cells were segmented from each experiment, translating to more than 13 billion numerical cell-level image measurements, which were stored in the database. For each protein, the four GFP and four red fluorescent protein (RFP) micrographs, along with the corresponding overlay GFP-RFP images, are available for visualization and download through CYCLoPs as lower resolution JPEG files.

### Quantitative scoring of protein abundance

Protein abundance was extrapolated from the mean GFP intensity. For each cell, we calculated the ratio of integrated GFP intensity measured within the area defined by the segmented cell boundary, divided by the segmented area of the cell. The mean GFP intensity (*I*_g_) of the protein was taken as the arithmetic mean of these ratios. The *I*_g_ measurements from our wild-type screen were highly correlated with protein abundance measurements from other techniques, namely flow cytometry, western blot analysis, and mass spectrometry (Chong *et al.* 2015). For each strain, protein abundance changes (∂PL) were calculated as the fold-change, *i.e.*, *I*_g_ in the presence of treatment/mutant over *I*_g_ in wild type. The *I*_g_ and ∂PL values for every protein in all conditions are readily searchable in CYCLoPs.

### Constructing the ensemble classifiers for quantification of subcellular localizations at single-cell level

Previous studies have shown that combining decisions from multiple computational classifier instances—a so-called “ensemble” strategy—can improve the predictive accuracy of the classification (Gashler *et al.* 2008; Rokach 2010). The ensemble approach is particularly useful in boosting the performance of weak learners and has been used in recent genomic studies (Chen *et al.* 2011; Reboiro-Jato *et al.* 2013). We constructed an ensemble of classifiers—ensLOC—to accurately assign each yeast cell to one or more of 16 pre-defined subcellular localization classes based on its morphological features. Our approach allows proteins to localize to more than one compartment or to remain unclassified, based on our 16 predefined morphologic classes. For every segmented cell in the compendium, ensLOC generated a 16-element vector, where each element is an independent assessment of the cell’s membership in a localization class.

**Figure 1:**
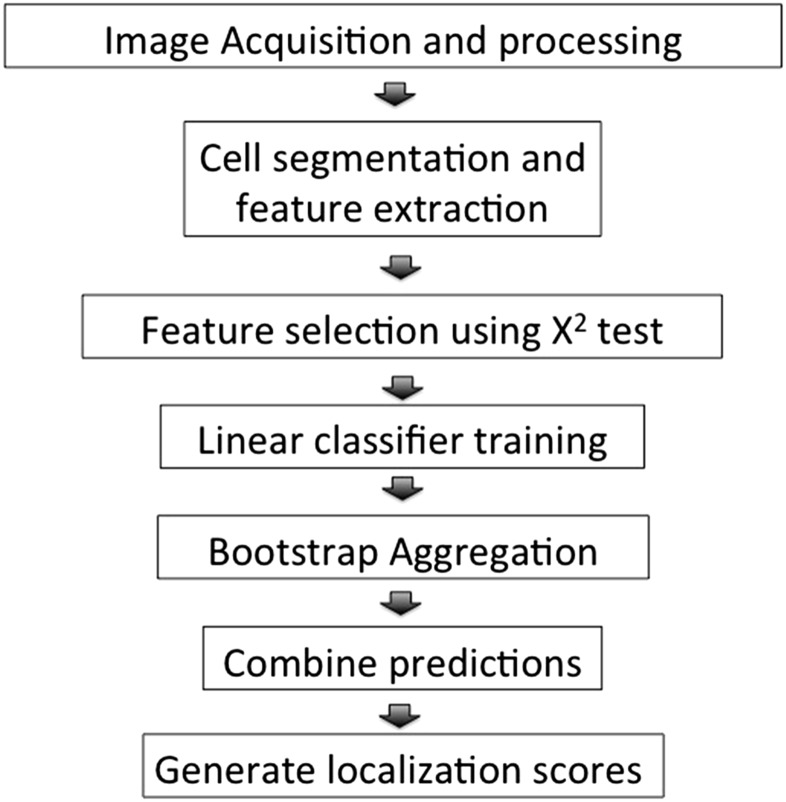
Overview of the ensLOC framework for quantifying subcellular localization of yeast proteins.

**Figure 2:**
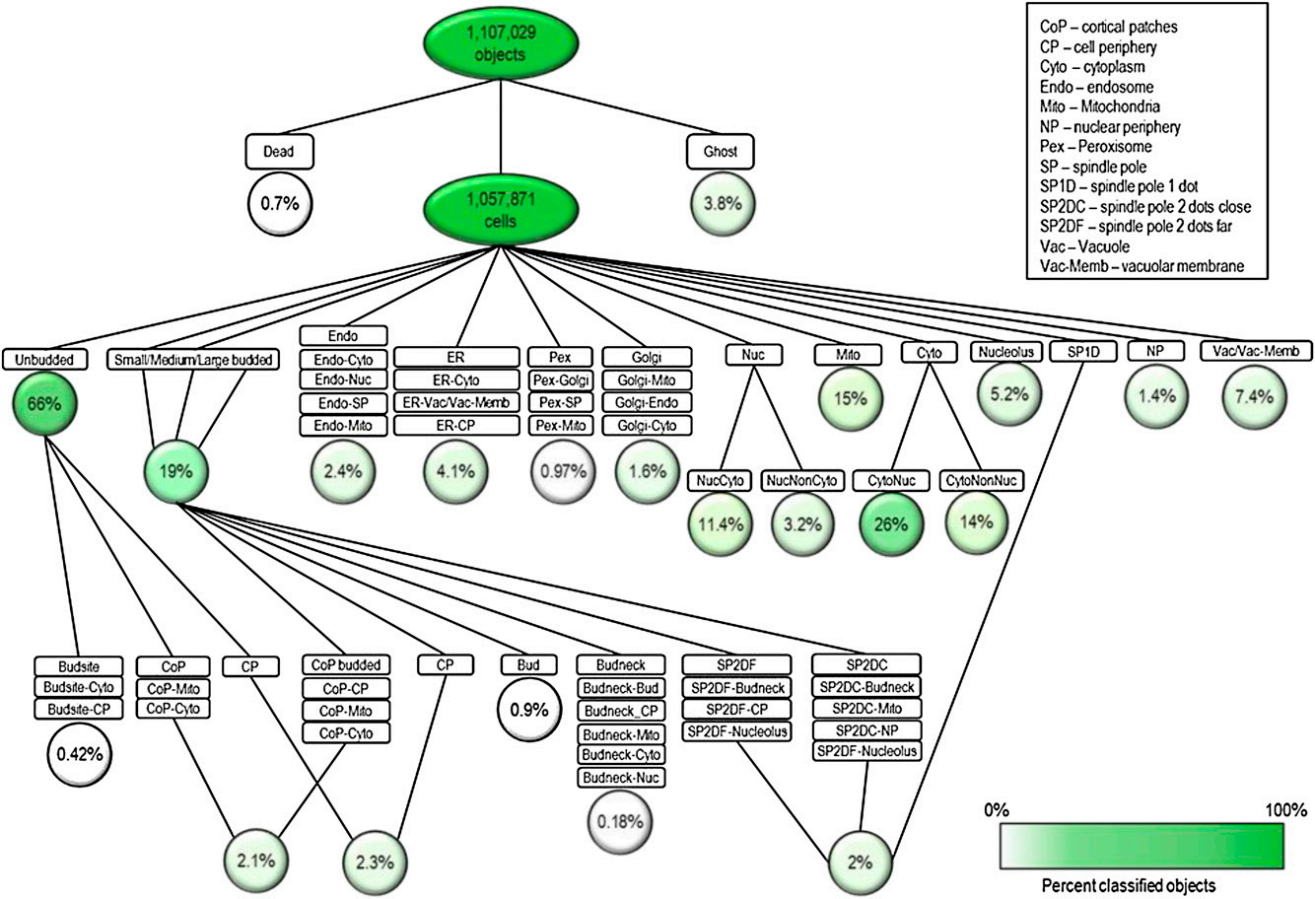
Diagram illustrating the ensemble of 60 binary classifiers for protein localization and quantification (modified from Chong *et al.* 2015). Only cell images that were not filtered by the quality-control classifiers for dead cells and “ghost” objects were further classified. All cells were first classified into different cell-cycle stages using the unbudded and budded classifiers. The rest of the ensemble is organized into 20 subgroups. For some classifier groups, *e.g.*, Cortical Patches and Cell Periphery, budded and unbudded cells were separately tested. The results from each subgroup of binary classifiers *e.g.*, CoP, CoP-Mito, and CoP-Cyto, were consolidated with Bagging. The circles denote the percentage of 1,057,871 cells in the wild-type WT1 experiment that were assigned to each localization class, with darker green indicating a greater percentage.

The ensLOC framework comprises several steps (Figure 1). We first segmented the cells from micrographs obtained from our wild-type screen. A total of 430 image features, including area, shape, intensity, texture, and Zernike moments (projections of image functions based on a set of orthogonal Zernike polynomials; Teague 1979) were extracted from the segmented cells. For each classifier, we used the χ^2^ test of independence (Liu and Setiono 1995) to select features that best discriminated the positive from the negative training instances. The filtered features were then used as input to construct the linear Support Vector Machine classifier (Platt 1998). Seventy thousand instances of cell images representative of the morphological signatures of 16 subcellular localizations were handpicked. The distribution of these training instances is shown in Figure 2 and Table 2. 

**Table 2:** The 60 binary classifiers used in the ensLOC framework

Classifier ID	Name of Binary Classifier	No. of Positive Training Objects	No. of Negative Training Objects-	Validation Using 10-fold Cross-Validation		Visual Inspection Recall
				Recall	Precision	
Quality control						
1.1.1	*DEAD*	960	1541	0.986	0.995	
1.1.2	*GHOST*	1840	2398	0.995	1	
Budded or Unbudded						
2.1.1	*UNBUDDED*	1095	1582	0.997	0.984	
2.1.2	*SMALLBUDDED*	434	733	0.952	0.948	
2.1.3	*LARGEMEDIUMBUDDED*	727	1508	0.985	0.986	
3.1 Cytoplasm						
3.1.1	*CYTOPLASM*	3493	4285	0.979	0.966	∼95%
3.1.2	*CYTOPLASMNOTNUCLEAR*	2075	1419	0.915	0.842	>95%
3.2 Endosome						
3.2.1	*ENDOSOME*	2245	4730	0.826	0.912	<70%
3.2.2	*ENDOSOME_CYTOPLASM*	2245	3493	0.977	0.995	
3.2.3	*ENDOSOME_NUCLEI*	2245	5612	0.995	0.999	
3.2.4	*ENDOSOME_SPINDLEPOLE*	2245	3397	0.963	0.986	
3.2.5	*ENDOSOME_MITOCHONDRIA*	2245	6315	0.899	0.967	
3.3 ER						
3.3.1	*ER*	5274	4259	0.977	0.919	<80%
3.3.2	*ER_CYTOPLASM*	5274	3493	0.97	0.965	
3.3.3	*ER_VACUOLEVACUOLARMEMBRANE*	5274	3893	0.976	0.958	
3.3.4	*ER_CELLPERIPHERY*	5274	4059	0.996	0.996	
3.4 Golgi						
3.4.1	*GOLGI*	1994	1838	0.964	0.908	>80%
3.4.2	*GOLGI_MITOCHONDRIA*	1994	6315	0.809	0.968	
3.4.3	*GOLGI_ENDOSOME*	1994	2245	0.919	0.934	
3.4.4	*GOLGI_CYTOPLASM*	1994	3493	0.996	0.999	
3.5 Mitochondria						
3.5.1	*MITOCHONDRIA*	6315	7894	0.894	0.884	>85%
3.6 Nuclear Periphery						
3.6.1	*NUCLEARPERIPHERY*	2668	4367	0.94	0.96	∼70%
3.7 Nucleus						
3.7.1	*NUCLEI*	5612	6881	0.977	0.956	>80%
3.7.2	*NUCLEINOTCYTOPLASM*	1398	989	0.99	0.93	>80%
3.8 Nucleolus						
3.8.1	*NUCLEOLUS*	3882	5332	0.926	0.948	>85%
3.9 Peroxisome						
3.9.1	*PEROXISOME*	1256	2099	0.849	0.922	<70%
3.9.2	*PEROXISOME_GOLGI*	1256	1993	0.928	0.971	
3.9.3	*PEROXISOME_SPINDLEPOLE*	1256	3397	0.965	0.995	
3.9.4	*PEROXISOME_MITOCHONDRIA*	1256	6315	0.814	0.981	
3.10 Vacuole/Vacuolar Membrane						
3.10.1	*VACUOLEVACUOLARMEMBRANE-COMBINED*	3893	3352	0.926	0.898	>80%
3.10.2	*VACUOLE_VACUOLARMEMBRANE*	2224	1846	0.92	0.845	>80% VAC, 65% VAC membrane
3.11 Cortical Patches						
3.11.1	*CORTICALPATCHESUNBUDDED*	1813	1279	0.964	0.877	∼70%
3.11.2	*CORTICALPATCHESUNBUDDED_CYTOPLASM*	1813	1661	0.994	0.996	
3.11.3	*CORTICALPATCHESUNBUDDED_MITOCHONDRIA*	1813	4440	0.95	0.984	
3.11.4	*CORTICALPATCHESBUDDED*	1345	2171	0.928	0.936	75%
3.11.5	*CORTICALPATCHESBUDDED_CELLPERIPHERY*	1345	1059	0.994	0.988	
3.11.6	*CORTICALPATCHESBUDDED_MITOCHONDRIA*	1345	1875	0.981	0.986	
3.11.7	*CORTICALPATCHESBUDDED_CYTOPLASM*	1345	1022	0.987	0.988	
3.12 Bud						
3.12.1	*BUD*	1619	1691	0.937	0.905	>70%
3.13 Budneck						
3.13.1	*BUDNECK*	2170	3095	0.947	0.942	>70%
3.13.2	*BUDNECK_BUD*	2170	1619	0.962	0.946	
3.13.3	*BUDNECK_CELLPERIPHERY*	2170	1059	1	0.994	
3.13.4	*BUDNECK_MITOCHONDRIA*	2170	1875	0.99	0.98	
3.13.5	*BUDNECK_CYTOPLASM*	2170	1022	0.987	0.98	
3.13.6	*BUDNECK_NUCLEI*	2170	1313	1	0.996	
3.14 Budsite						
3.14.1	*BUDSITE*	453	637	0.982	0.961	>80%
3.14.2	*BUDSITE_CYTOPLASM*	453	4955	0.943	0.992	
3.14.3	*BUDSITE_CELLPERIPHERY*	453	359	0.996	0.992	
3.15 Cell Periphery						
3.15.1	*CELLPERIPHERYUNBUDDED*	2269	858	0.989	0.98	>95%
3.15.2	*CELLPERIPHERYBUDDED*	1059	1688	0.981	0.991	>85%
3.16 Spindle Pole						
3.16.1	*SPINDLEPOLETWODOTFARBUDDED*	416	966	0.938	0.965	>70%
3.16.2	*SPINDLEPOLETWODOTFARBUDDED_BUDNECK*	416	2170	0.913	0.997	
3.16.3	*SPINDLEPOLETWODOTFARBUDDED_NUCLEARPERIPHERY*	416	492	1	0.996	
3.16.4	*SPINDLEPOLETWODOTFARBUDDED_NUCLEOLUS*	416	1109	0.99	0.995	
3.16.5	*SPINDLEPOLETWODOTCLOSEBUDDED*	306	1016	0.905	0.97	∼80%
3.16.6	*SPINDLEPOLETWODOTCLOSEBUDDED_BUDNECK*	306	2170	0.899	0.995	
3.16.7	*SPINDLEPOLETWODOTCLOSEBUDDED_MITOCHONDRIA*	306	1875	0.974	0.996	
3.16.8	*SPINDLEPOLETWODOTCLOSEBUDDED_NUCLEARPERIPHERY*	306	492	0.993	0.996	
3.16.9	*SPINDLEPOLETWODOTCLOSEBUDDED_NUCLEOLUS*	306	1109	0.98	0.988	
3.16.10	*SPINDLEPOLEONEDOT*	2675	3676	0.974	0.983	70%

In total, approximately 70K handpicked cell images (objects) were used to train the classifiers. “No. of positive training objects” refers to cells which belong to the targeted class and “No. of negative training objects” refer to cells not belonging to the targeted class. For example, to construct the “DEAD” cells classifier, 960 images of dead cells were used as positive training objects and 1541 images of non-dead cells from across all 16 localization classes were used as negative training objects. The first number of the classifier ID reflects the level and therefore the sequence at which the classifier was applied. For instance, all cell images were first tested using the “DEAD” cells classifier to eliminate dead cells from further classification to the 16 localization classes, and only cells that were tested positive in the level 2 “SMALLBUDDED” and “LARGEMEDIUMBUDDED” classifiers would be further classified by the “BUDNECK” classifier. The accuracy of the classifiers was validated computationally using 10-fold cross-validation and manually using visual inspection of 500 random positive cells. Recall = True positives/(True positives + False negatives); Precision = True positives/(True positives + False positives). ER, endoplasm reticulum.

Predictions for each subcellular localization class were obtained through combining the predicted results of a set of binary classifiers (Figure 2). A binary classifier classifies elements of a given test set into only two groups. For example, a cell is assigned to the endoplasm reticulum (ER) class if it is assigned to ER in the ER-all, ER-Cytoplasm and ER-Vacuole/Vacuolar Membrane, and ER-Cell Periphery binary classifiers (Table 2). Our objective with this approach was to reduce misclassifications among subcellular localization classes with overlapping morphological patterns. In addition, an improvement of predictive accuracy was achieved through bootstrap aggregation (bagging) (Breiman 1996), specifically by plurality voting. We generated 25 bag classifiers, each from 1000 random training instances (500 positive and 500 negative samples) with replacements. Decisions for the localization assignments of a binary classification step were reconciled from these 25 bag classifiers. Thus, in total the localization assignment for a segmented cell was determined through plurality voting of more than 1000 classifiers (25 bags × 60 binary classifiers). Because the computation was time intensive, we modified the algorithm to enable parallelized execution on a computer cluster. Both 10-fold cross-validation and visual inspection of random samples were independently conducted for each localization class to validate the accuracy of the classifier (Table 2).

**Figure 3:**
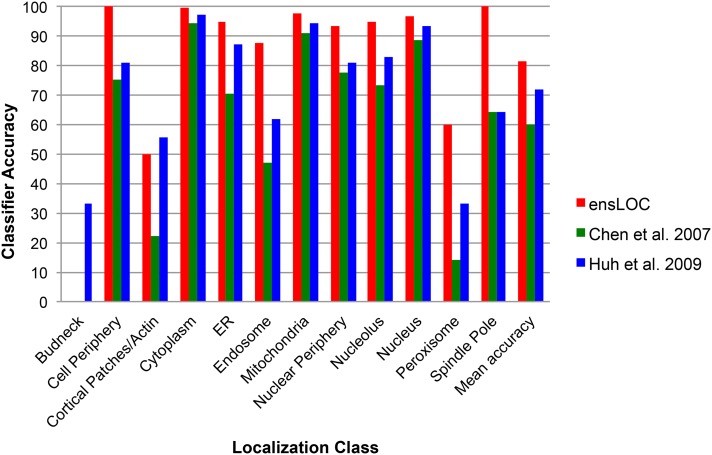
Classifier accuracy of the ensLOC framework. The accuracy of the ensLOC framework (red) in assigning protein localization to 12 different subcellular compartments (X-axis) is compared with two other automated classification methods (Chen *et al.* 2007, green; Huh *et al.* 2009, blue).

We benchmarked our computationally derived localization assignments for one of our wild-type screens, WT1 (Chong *et al.* 2015), to visually assigned localization annotations from YGFP (Huh *et al.* 2003) and found 94% agreement among the set of 1097 proteins assigned to a single location by both methods (Chong *et al.* 2015). We also compared our computationally derived localization assignments with assignments made using other computational methods with images from YGFP (Chen *et al.* 2007; Huh *et al.* 2009). The ensLOC framework achieved greater mean accuracy (overlap proteins divided by number of proteins identified in YGFP) for proteins identified as having a single localization in both data sets. The ensLOC framework attained a mean classifier accuracy of 81%, an improvement of up to 20% across 12 subcellular localization classes defined in both methods (Figure 3). It is also worth noting that, unlike other methods, the ensLOC framework for quantifying subcellular localization does not restrict a protein to a single localization class.

### Quantitative scoring of subcellular localization using the ensLOC framework

In our automated imaging pipeline (Chong *et al.* 2015), an experiment generally produced more than a million segmented cells, among which approximately 5% were of inadequate quality. These poor quality cell images were removed using a quality control step with classifiers designed to identify dead and “ghost” cells. “Ghost” objects are artifacts that get recognized in the background of an image, as a consequence of noise being recognized as signal and result in segmentation of a region containing no cells. The ensLOC framework was then applied independently to each filtered cell; that is, protein localization in each cell was predicted for up to 60 binary classifiers, where each classifier determined if a cell should be assigned positively or negatively to the class based on its morphological features. For example, the ER-Cytoplasm binary classifier determined whether a cell harbored the phenotypic signatures of ER localization class (positive) or Cytoplasm localization class (negative). To determine the subcellular localization assignment profile of a GFP-tagged protein at the single-cell level, we calculated the proportion of labeled cells that were assigned to each of the 16 subcellular localization classes. The localization profile of a protein is thus represented as a 16-element vector, where each element (“LOC-score”) reflects the proportion of “classifiable” cells (that is, assigned to at least one localization class) that are assigned to a specific localization class.

To identify changes in subcellular localization for each protein following genetic or environmental perturbation, we assessed the statistical significance of the difference between the proportion of cells with a given localization in a condition (genetic/chemical perturbation) and the proportion of cells in wild type by using a metric we designated a z-LOC score. Cutoffs for significant localization changes were determined by fitting a “background” normal Gaussian model and a uniform “outlier” density model to the z-LOC score distribution. Cutoffs were chosen such that the number of true “outliers” was optimized (Chong *et al.* 2015). The LOC-scores and z-LOC scores are readily searchable in CYCLoPs.

### Database system construction

The relational database schema of CYCLoPs was developed to provide central storage and querying of different types of data generated from our systematic yeast imaging experiments. Our goal was to optimize the efficient and scalable querying of the micrographs, the LOC-score and z-LOC score profiles, and the abundance *I*_g_ and δPL score profiles of all proteins and conditions surveyed (database schema available through the CYCLoPs online documentation). The backend of CYCLoPs features a *mySQL* relational database management system, which comprises more than 100 experiment-specific tables, and the front-end web interface is hosted on an Apache 2.0 web server. The web interface was developed using a combination of HTML, CGI Perl, Perl DataBase Interface, Cascading Style Sheets, Javascript and R plotting libraries.

### Database utility

#### System interfaces and visualization:

CYCLoPs is primarily accessible via a Web interface, with a focus on providing easy and efficient access to a genome-wide database of quantitative descriptors of protein dynamics, and to assist biologists in experiment planning and hypothesis generation. A number of query and visualization tools are included in CYCLoPs version 1.0, including two custom-made micrograph viewers.

**Figure 4:**
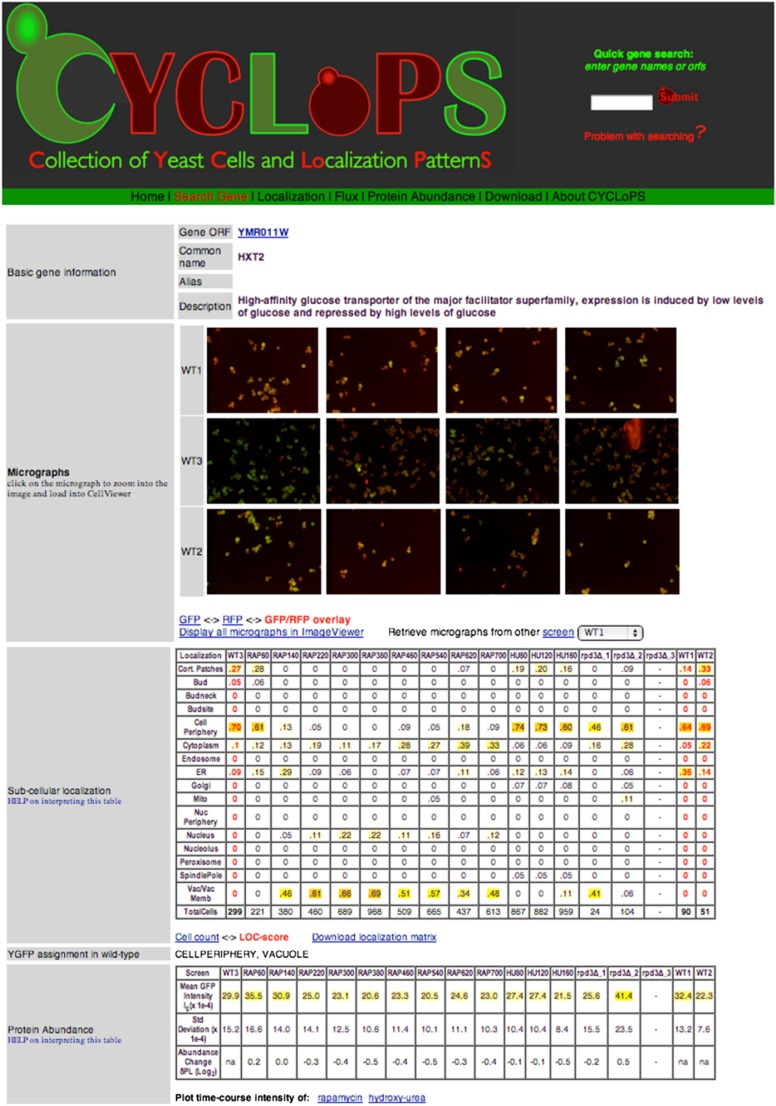
Screen shot of sample search and result page generated by CYCLoPs is shown. A query of the Hxt2 protein produces images of yeast cells from three wild-type screens (WT1, WT2, WT3). The tables list numerical measurements of protein abundance (*I*_g_), protein abundance changes (δPL), subcellular localization (LOC-scores), and subcellular localization changes (zLOC-scores) with localizations from the WT screens shown in red. The scores are highlighted using a color scale from white to yellow to red to allow the viewer to identify variances in a range of values with a quick glance and do not represent significance values.

Various search options are available. Search options for proteins include protein name, common name, alias, and ORF. A protein-centric search returns a general description, abundance scores, and localization scores for the best matching protein across all screens. For example, a quick search of “Hxt2” returns a report displaying: (1) the micrographs from three wild-type screens; (2) a subcellular localization table depicting the LOC-scores of Hxt2 for each of 16 localization classes (rows) across all screens (columns); (3) a protein abundance table showing the abundance *I*_g_ and changes δPL across the screens and; (4) a subcellular localization change table showing the localization changes, z-LOCs, for each localization class across all screens (Figure 4). Search results and images may be downloaded and direct visualization of each individual cell in the compendium is available through the companion Image Viewer and Cell Viewer.

#### Image and cell viewer:

**Figure 5:**
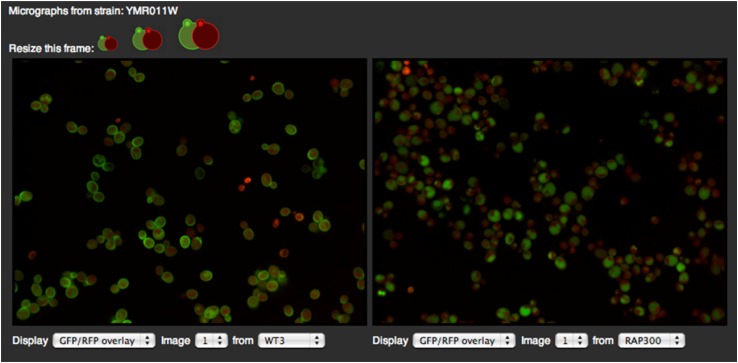
ImageViewer showing micrographs of a wild-type strain expressing Hxt2-GFP after growth in standard medium (left) and 300 min after treatment with rapamycin (RAP300, right).

**Figure 6:**
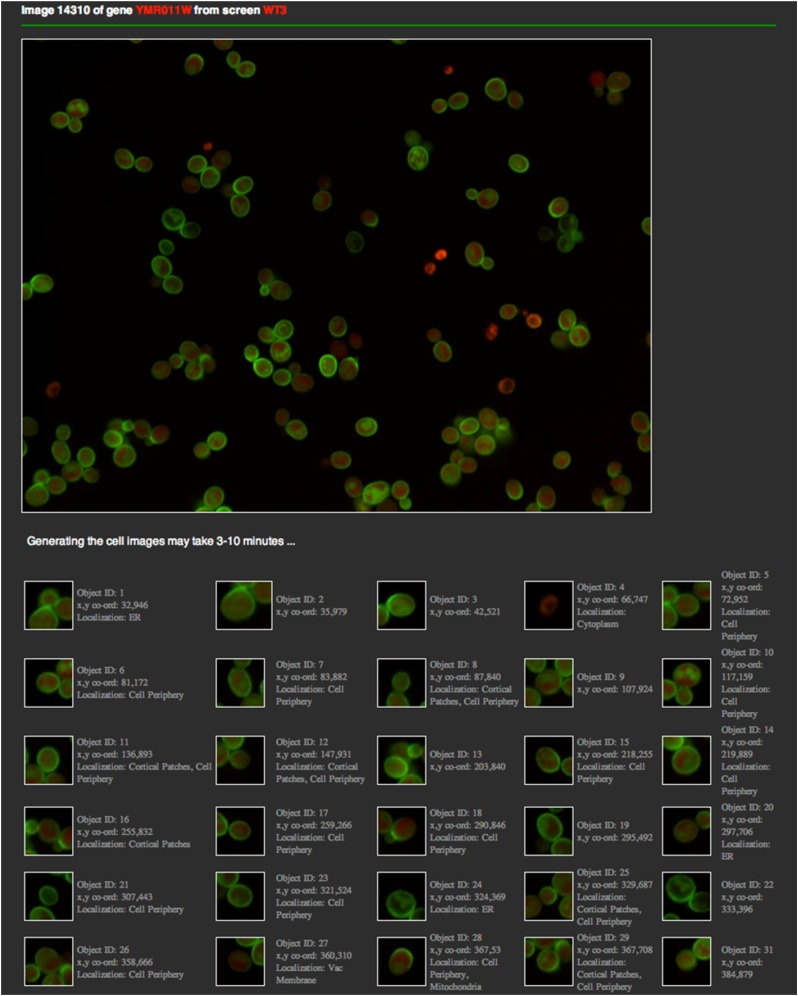
Cell viewer for inspecting individual cells in a micrograph.

The Image Viewer facilitates visual inspection of pairs of micrographs. Users can toggle between 18 screens, four images per screen, and three image channels (RFP/GFP/GFP-RFP overlay). This tool is particularly useful for visual inspection of morphologic changes. For example, Figure 5 shows internalization of Hxt2, a glucose transporter, in response to rapamycin treatment. Cells in the left micrograph (from a WT screen) display morphologic patterns that define a cell-periphery localization of Hxt2, whereas most cells in the right micrograph (after 300 min of rapamycin treatment) exhibit an obvious localization to vacuole/vacuolar membrane. The Cell Viewer provides a detailed view of a specified micrograph by cropping it into individual cells (Figure 6). The position coordinates of each cell image were obtained from the cell segmentation routine in our image analysis program. The localization labels of the cell were determined using our ensemble classifiers.

#### Single cell abundance measurements and localization assignments:

Both localization and abundance of a protein may vary in individual cells in a population (Chong *et al.* 2015), and analysis of single cells can give important information about cell-cycle events and stochasticity. Because all of our data were acquired at the level of the individual cell, we are able to provide a function that allows the user to download abundance and localization data for single cells. This function may be found at http://cyclops.ccbr.utoronto.ca/DOWNLOAD/Download.html. For a selected image in our compendium, this function generates a text file with the following columns:

Object ID, X-coordinate, Y-coordinate, GFP Intensity (Ig), Localization.

#### Querying top protein abundance and localization changes:

Users also may retrieve top-ranked proteins that are transported toward or away from any of the 16 subcellular compartments included in the database or that exhibit increase/decrease in protein abundance in the presence of drug treatment or gene deletion. Search results and images may be downloaded and direct visualization of each object/cell in the compendium is available through the companion Image Viewer and Cell Viewer.

We have generated a compendium of RFP/GFP micrographs and quantitative measurements of subcellular localization and abundance changes covering ∼71% of the yeast proteome in response to genetic and chemical perturbations. To make this novel compendium available and useful to the research community, we have developed a web-based query system for accessing, visualizing and analyzing the data.

CYCLoPs is intended to be an active resource for quantitative genome-wide localization and abundance measurements of *S. cerevisiae* made in multiple genetic backgrounds and following different chemical treatments. Future enhancements of CYCLoPs will involve automation processes for experimental updates. In revised versions of CYCLoPs, we hope to integrate data from other external sources for on-the-fly cross-dimensional comparisons and visualization. CYCLoPs is tightly integrated with our experimental and scoring platform, and will house data from future experiments designed to test the response of the yeast proteome to a variety of chemical and environmental perturbations.
